# Clinical and Molecular Evidence of ABCC11 Protein Expression in Axillary Apocrine Glands of Patients with Axillary Osmidrosis

**DOI:** 10.3390/ijms18020417

**Published:** 2017-02-15

**Authors:** Yu Toyoda, Tappei Takada, Tsuneaki Gomi, Hiroshi Nakagawa, Toshihisa Ishikawa, Hiroshi Suzuki

**Affiliations:** 1 Department of Pharmacy, The University of Tokyo Hospital, 7-3-1 Hongo, Bunkyo-ku, Tokyo 113-8655, Japan; tappei-tky@umin.ac.jp (T.T.); suzukihi-tky@umin.ac.jp (H.S.); 2 Gomi Clinic, 1-10-12, Hyakunin-cho, Shinjyuku-ku, Tokyo 169-0073, Japan; gomi@gomiclinic.com; 3 Department of Applied Biological Chemistry, Graduate School of Bioscience and Biotechnology, Chubu University, 1200 Matsumoto-cho, Kasugai 487-8501, Japan; hnakagaw@isc.chubu.ac.jp; 4 RIKEN Center for Life Science Technology, 1-7-22, Suehiro-cho, Tsurumi-ku, Yokohama 230-0045, Japan; toshihisa.ishikawa.r@gmail.com

**Keywords:** armpit, body odor, MRP8, protein stability, single nucleotide polymorphism, transporter

## Abstract

Accumulating evidence suggests that the risk of axillary osmidrosis is governed by a non-synonymous single nucleotide polymorphism (SNP) 538G>A in human *ATP-binding cassette C11* (*ABCC11*) gene. However, little data are available for the expression of ABCC11 protein in human axillary apocrine glands that produce apocrine sweat—a source of odor from the armpits. To determine the effect of the non-synonymous SNP *ABCC11* 538G>A (G180R) on the ABCC11 in vivo, we generated transiently ABCC11-expressing transgenic mice with adenovirus vector, and examined the protein levels of each ABCC11 in the mice with immunoblotting using an anti-ABCC11 antibody we have generated in the present study. Furthermore, we examined the expression of ABCC11 protein in human axillary apocrine glands extracted from axillary osmidrosis patients carrying each *ABCC11* genotype: 538GG, GA, and AA. Analyses of transiently ABCC11-expressing transgenic mice showed that *ABCC11* 538G>A diminishes the ABCC11 protein levels in vivo. Consistently, ABCC11 protein was detected in the human axillary apocrine glands of the 538GG homozygote or 538GA heterozygote, not in the 538AA homozygote. These findings would contribute to a better understanding of the molecular basis of axillary osmidrosis.

## 1. Introduction

Strong or specific body odors may be perceived as unpleasant, and tackling body odor issues as part of daily grooming activities is a common practice in modern society [1,2]. While body odor is influenced by various physiological conditions and skin flora, it also has a genetic basis. In humans, axillary odor is often recognized as a distinctive malodor [3]. In particular, axillary osmidrosis (AO) could affect an individual’s social life because of the associated strong odors and profuse sweating from the armpit apocrine glands [1,3,4,5]. Recently, we and other research groups independently revealed the genetic background of AO [1,6,7,8,9].

Accumulating evidence suggests that AO risk is governed by a non-synonymous single nucleotide polymorphism (SNP) 538G>A (rs17822931: Gly180Arg) in the human *ATP-binding cassette C11* (*ABCC11*) gene, which is also a determinant of human earwax type [10]. The 538G allele has been found to be intimately associated with the high-secretory phenotypes of the apocrine gland, leading to a risk of AO and the wet type of earwax; the 538A allele was associated with the low-secretory phenotypes. Our in vitro study revealed that the ABCC11 wild-type (WT, 538G genotype) protein is matured as a glycoprotein [8]. In contrast, the variant ABCC11 protein with Arg180 (R180) resulting from the 538A genotype is subjected to endoplasmic reticulum-associated protein degradation (ERAD), which results in the loss of function [8]. However, to date, little is known of the ABCC11 protein expression levels in human axillary apocrine glands.

In this study, we determined the effect of the SNP *ABCC11* 538G>A on ABCC11 protein levels in vivo. For this purpose, we generated an anti-human ABCC11 antibody and studied the transgenic mice that transiently expressed human ABCC11 cDNA with either the 538G or 538A genotype. Furthermore, we immunologically detected ABCC11 protein expression in the axillary apocrine glands of humans carrying each *ABCC11* genotype.

## 2. Results

### 2.1. Generation and Validation of Anti-ABCC11 Antibody

First, we generated a polyclonal antibody against human ABCC11 (09YT) and validated its specificity to human ABCC11 (Figure S1). In order to generate a polyclonal antibody against human ABCC11, the intracellular domain (A747 to H795) located between transmembrane domains 6 and 7 of the ABCC11 protein (Figure S1) was selected as an epitope. Three kinds of keyhole limpet hemocyanin-conjugated synthetic oligopeptides that covered the epitope region (NH_2_–C+AKIAEKPKVESQALATSLEESLNGNAVPE–COOH, NH_2_–C+SLEESLNGNAVPEHQLTQEEEME EG–COOH, and NH_2_–C+HQLTQEEEMEEGSLSWRVYHH–COOH) were used to immunize a rabbit and to purify the rabbit antiserum by using an epitope peptide-conjugated affinity column. The specificity of the obtained antibody for human ABCC11 (09YT) was validated before use (Figure S1).

### 2.2. Generation and Analysis of Transiently ABCC11-Expressing Transgenic Mice

**Figure 1:**
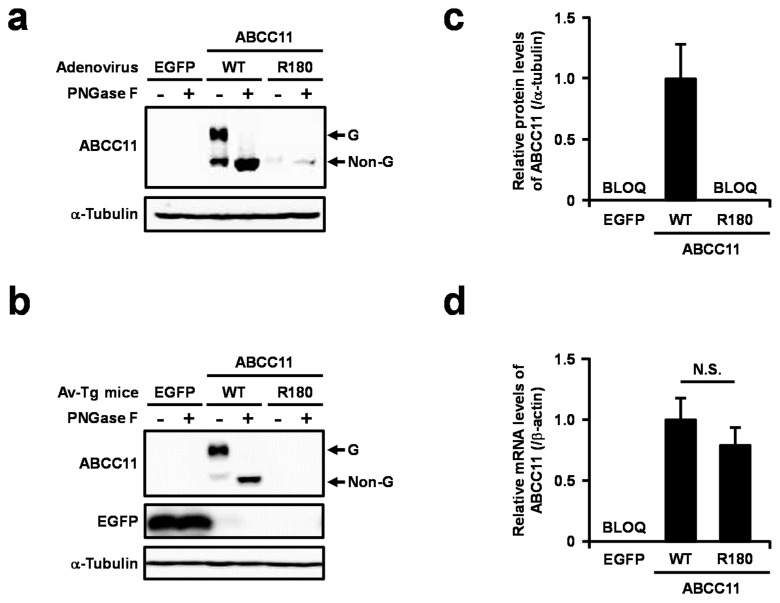
Effect of single nucleotide polymorphism (SNP) 538G>A (G180R) on the expression of the ATP-binding cassette C11 (ABCC11) protein in transiently transgenic mice. (**a**) Immunoblotting detection of the ABCC11 protein expressed in Hepa1-6 cells. The function of the ABCC11 wild-type (WT) expressed by the adenovirus vector was confirmed using an in vitro vesicle transport experiment (Figure S2). The immuno-reactive band corresponding to the glycosylated form (G) of the ABCC11 protein disappeared after peptide *N*-glycosidase F (PNGase F) treatment. Non-G: non-glycosylated form; α-Tubulin: a loading control; (**b**) Maturation status and expression of the ABCC11 protein in the liver of adenovirus-infected transiently transgenic (Av-Tg) mice; (**c**) Densitometric analysis of ABCC11 protein levels in each group of Av-Tg mice. *n* = 4; (**d**) ABCC11 mRNA levels in each group of Av-Tg mice. The mRNA levels were normalized to the WT (control) level. *n* = 5. β-Actin: a housekeeping gene for internal control. Data are expressed as mean ± S.E.M. N.S., not significantly different between groups. BLOQ, below limit of quantification.

Next, we prepared adenovirus vectors to express ABCC11 WT and R180 in vivo. Administered adenoviruses generally accumulate in the liver of experimental animals, resulting in hepatic expression of the transgene. Therefore, prior to the in vivo experiments, we confirmed the adenovirus-mediated expression of ABCC11 in vitro by using murine hepatic Hepa1-6 cells (Figure 1a). We chose mice as the infectious host because they do not have the *Abcc11* gene [11]. As expected, the ABCC11 WT was detected in the mature form as a glycoprotein, while the matured R180 variant was not detected (Figure 1a). The R180 variant protein levels were considerably lower than the WT protein levels. These results were consistent with a previous study demonstrating that the R180 protein was rapidly eliminated from cells through ERAD [8].

To examine the effect of the *ABCC11* 538G>A on the expression of ABCC11 in vivo, we used adenoviruses to generate transiently transgenic mice that expressed the ABCC11 WT and R180 variant, and performed immunoblotting using the anti-ABCC11 antibody (09YT). In the liver of the mice administered the ABCC11 WT-expressing adenovirus, most of the ABCC11 protein was detected as a mature form that was sensitive to the digestion of *N*-glycans with *N*-glycosidase treatment (Figure 1b,c). In the case of R180 variant, ABCC11 protein was not detected in the liver, nor in control mice. At that time, there were no significant differences in the mRNA levels of each ABCC11 in the liver (Figure 1d). These results indicate that the ABCC11 R180 protein underwent rapid degradation in vivo as compared to the WT protein, probably resulting in the loss of function of the R180 variant as well as in vitro.

### 2.3. Expression of ABCC11 WT Protein in Axillary Apocrine Glands Derived from AO Patient

**Figure 2:**
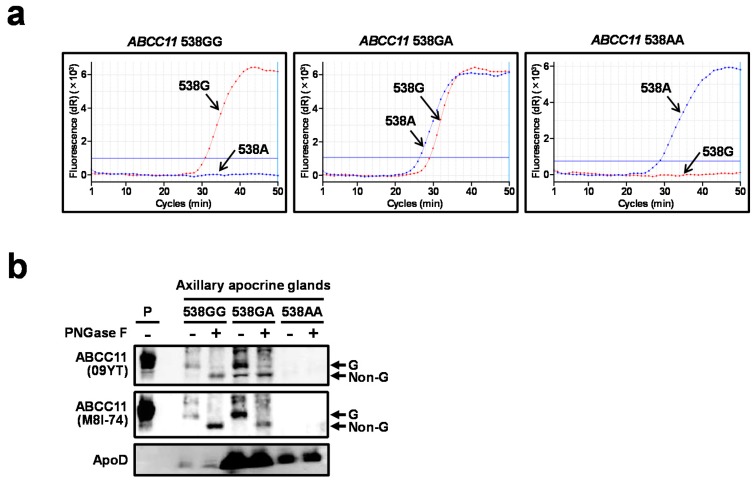
Expression of ABCC11 protein in the extracted subcutaneous tissues containing axillary apocrine glands in axillary osmidrosis (AO) patients carrying *ABCC11* 538G. (**a**) Time-dependent signal increase for the genotyping at *ABCC11* 538G>A by the SmartAmp method. Changes in fluorescence intensity of SYBR Green I dye with allele-specific primers (red: 538G; blue: 538A) were monitored under isothermal conditions (60 °C, 1 min/cycle) by using an Mx3000P QPCR system (Agilent Technologies, Santa Clara, CA, USA); (**b**) Immunoblotting analysis of the axillary apocrine glands extracted from the AO patients. The same amount of protein was loaded in each SDS-PAGE lane. Successful detection of ABCC11 and ApoD in the 538GG and GA lanes should ensure that enough amount of whole tissue lysate sample for the qualitative evaluation of ABCC11 protein level was loaded in the 538AA lanes where the middle levels of ApoD in a set of measurements were determined. ABCC11 protein expression was determined by using anti-ABCC11 antibodies 09YT and M8I-74. ApoD: apolipoprotein D, an apocrine gland marker. P: positive control; whole cell lysate of ABCC11 WT-expressing 293A cells. G: glycosylated, Non-G: non-glycosylated form of the ABCC11 protein.

We next examined the expression of the ABCC11 protein in the axillary apocrine glands extracted from AO patients for whom the *ABCC11* genotype was determined by using the SmartAmp-based method [1,12] (Figure 2a). Owing to surgical limitations and the risk of extracting subcutaneous tissues other than apocrine glands, we determined the expression of apolipoprotein D protein—an apocrine gland marker [13]—to confirm that the extracted tissues contained apocrine glands. In all clinical samples, we successfully detected the apolipoprotein D protein by difference in the strength of detected signals (Figure 2b), which must be affected by the experimental limitations and/or the individual differences in the apolipoprotein D expression. In the samples carrying *ABCC11* 538GG and 538GA, immuno-reactive signals corresponding to the ABCC11 protein were detected by immunoblotting, and these signals were sensitive to *N*-glycosidase treatment (Figure 2b). In contrast, there was no immuno-reactive signal corresponding to ABCC11 in the apocrine glands carrying *ABCC11* 538AA. Similar results were obtained by using another functional anti-ABCC11 antibody (M8I-74) which is commercially available (Figure 2b) [11]. These twice-confirmed results indicated that the ABCC11 WT protein is expressed as a mature form, and R180 protein has little expression in axillary apocrine glands.

## 3. Discussion

In the present study, we demonstrated that *ABCC11* 538G>A diminishes ABCC11 protein levels in vivo (Figure 1). The expression of ABCC11 mRNA in the transiently transgenic mice was driven by an exogenous cytomegalovirus promoter (which has strong transcriptional activity), and did not differ significantly between both genotypes. Nevertheless, the protein levels of the R180 variant were extremely lower than those of ABCC11 WT, suggesting that the endogenous ABCC11 protein is also affected by the SNP. Indeed, the ABCC11 protein was detected in the axillary apocrine glands of subjects with the GG homozygote and GA heterozygote, but not in those with the AA homozygote (Figure 2b). To the best of our knowledge, this is the first report on the use of immunoblotting for the detection of ABCC11 protein in axillary apocrine glands in humans.

Before concluding, the limitations and future perspectives of this study will be discussed. Unfortunately, we could not assess the difference about the odor rating and protein levels of ABCC11 in apocrine glands between 538GG and GA patients with AO, owing to the small number of samples in the present study. In order to reveal this issue, further investigation should be addressed in the future. Notably, in such a study, not Asian but European might be research-friendly population, since the allele frequency of *ABCC11* 538G is higher in European populations (0.875) than in Asian populations (0.110) according to the HapMap-CEU and HapMap-JPT data, respectively.

In conclusion, the findings in the present study deepen our understanding of ABCC11 biology and the molecular basis of AO. Considering the growing interest in the regulation of human body odors, complete elucidation of the molecular basis of AO should be carried out in future investigations.

## 4. Materials and Methods

### 4.1. Materials

A rabbit polyclonal antibody against the human ABCC11 protein (anti-ABCC11 antibody (09YT)) was raised by immunizing rabbit with synthetic oligo-peptides as described above. Other anti-ABCC11 antibodies—such as H-215 (sc-20969) and M8I-74 (ab91452)—were purchased from Santa Cruz Biotechnology, Inc. (Santa Cruz, CA, USA) and Abcam Inc. (Cambridge, MA, USA), respectively. Antibodies against α-tubulin (ab15246) and apolipoprotein D (ab108191) were obtained from Abcam Inc. Antibody against enhanced green fluorescent protein (EGFP) (A11122) was purchased from Life Technologies (Tokyo, Japan). A donkey anti-rabbit immunoglobulin G (IgG)-horseradish peroxidase (HRP)-conjugated antibody (NA934V) and a goat anti-rat IgG-HRP-conjugated antibody (NA935V) were purchased from GE Healthcare UK Ltd. (Buckinghamshire, UK). All the other chemicals used were commercially available and of analytical grade.

### 4.2. Cell Culture

Human embryonic kidney 293 (HEK293)-derived 293A cells and murine hepatocellular carcinoma-derived Hepa1-6 cells were maintained in Dulbecco’s Modified Eagle’s Medium (Nacalai Tesque, Inc., Kyoto, Japan) supplemented with 10% fetal bovine serum (Biowest, Nuaillé, France) and 1% penicillin-streptomycin (Nacalai Tesque, Inc.), 2 mM l-glutamine (Nacalai Tesque, Inc.), and 1× Non-Essential Amino Acid (Life Technologies) at 37°C in a humidified atmosphere of 5% (*v*/*v*) CO_2_ in air. Adenovirus infection was performed as described previously [11].

### 4.3. Construction of and Infection with Recombinant Adenovirus

Recombinant adenoviruses for the expression of the human ABCC11 WT (NCBI accession; NM_033151) and R180 variant were constructed and amplified in 293A cells as described previously [11]. An EGFP-expressing adenovirus (control) was derived from our previous study [14]. After the amplification, each adenovirus in the cell extract was purified by CsCl gradient ultracentrifugation; dialyzed with a solution containing 10 mM Tris (pH 7.5), 1 mM MgCl_2_, and 10% glycerol; and stored in aliquots at −80 °C until use. The adenovirus titer was then determined by using an Adeno-X™ Rapid Titer Kit (Clontech Laboratories, Inc., Palo Alto, CA, USA). To carry out functional validation of the ABCC11 WT produced by the adenovirus (Figure S2), an in vitro transport experiment was conducted as described below. Furthermore, after the treatment with MG132 (Calbiochem, Darmstadt, Germany)—a proteasome inhibitor, the generation of ABCC11 R180 protein from the adenovirus vector was confirmed by immunoblotting analysis (Figure S3). In addition, this result indicated that the ABCC11 R180 protein is degraded by proteasome-dependent pathway in normal condition.

### 4.4. Preparation of Plasma Membrane Vesicles and the In Vitro Transport Experiment

Membrane vesicles were prepared from 293A cells infected with the ABCC11-expressing or EGFP-expressing (control) adenovirus as described in our previous report [15]. Experiments to study the in vitro transport of [^3^H]estrone sulfate (E_1_S) (Perkin-Elmer Japan Co., Ltd., Tokyo, Japan)—an ABCC11 substrate [16,17]—into ABCC11-expressing and control vesicles were performed by using a rapid filtration technique [18,19] with a minor modification. In brief, the membrane vesicles (5 µg) were incubated with a reaction mixture containing 500 nM of [^3^H]E_1_S in the presence or absence of ATP for 10 min, and the radioactivity derived from the incorporated E_1_S was measured. In this transport experiment, the transport activity in each group was calculated as incorporated clearance (µL/mg protein/min = incorporated level of [^3^H]E_1_S (disintegrations per minute (DPM)/mg protein/min)/[^3^H]E_1_S level in the incubation mixture (DPM/µL)).

### 4.5. Animals

Wild-type C57BL/6J male mice were purchased from CLEA Japan (Tokyo, Japan). The mice used in the present study were 6–7 weeks of age, fed a standard diet and water ad libitum, and maintained under a 12-hour light and dark cycle as described previously [18,20]. Under inhalation anesthesia with isoflurane (Wako Pure Chemical Industries Ltd., Tokyo, Japan), the mice were intravenously administered with the adenoviruses (1 × 10^10^ infectious unit/20 g of body weight). Necropsy was performed 3 days after the infection, and livers were excised, weighed, rapidly frozen in liquid nitrogen, and stored at −80 °C until further processing. All animals were handled with humane care, and experiments were performed according to the methods approved by the Institutional Animal Care Committee of the University of Tokyo (No. H12-88).

### 4.6. Immunoblotting Analysis

Whole cell lysates were prepared as described previously [8]. Tissue homogenates (g of tissue/20 mL) were obtained in buffer A containing a protease inhibitor cocktail for general use (Nacalai Tesque) on ice using an ice-cold Physcotron homogenizer (Microtec Co., Ltd., Chiba, Japan) and centrifuged at 3000× *g* at 4°C for 20 min. The resulting supernatant was collected in a new tube, and the protein concentration was determined by using the BCA Protein Assay Kit (Pierce, Rockford, IL, USA). The whole cell lysates and tissue homogenates were treated with Peptide *N*-glycosidase F (PNGase F) (New England Biolabs, Inc., Ipswich, MA, USA) as described previously [21], and then subjected to immunoblotting analysis.

The prepared samples were mixed with the sodium dodecyl sulfate polyacrylamide gel electrophoresis (SDS-PAGE) sample buffer solution containing 10% 2-mercaptoethanol, electrophoretically separated on poly-acrylamide gels, and then transferred to Hybond^®^ ECL^TM^ nitrocellulose membrane (GE Healthcare UK Ltd.) by electroblotting at 15 V for 70 min. For blocking, the membrane was incubated in Tris-buffered saline containing 0.05% Tween 20 and 5% skim milk (TTBS-skim milk) at 4 °C overnight. Blots were probed with the anti-ABCC11 antibody (09YT, 1/1000 diluted in TTBS-skim milk; M8I-74, 1/200; H-215, 1/500), anti-α-tubulin antibody (1/1000), anti-EGFP antibody (1/1000), or anti-apolipoprotein D antibody (1/1000) followed by HRP-conjugated secondary antibodies. For clinical samples, the amount of each anti-ABCC11 antibody was doubled. HRP-dependent luminescence was developed with ECL^TM^ Prime Western Blotting Detection Reagent (GE Healthcare UK Ltd.) and detected using a luminescent image analyzer (Bio-Rad Laboratories, Tokyo, Japan) or a multi-imaging Analyzer Fusion Solo 4^TM^ system (Vilber Lourmat, Eberhardzell, Germany).

### 4.7. Quantification of mRNA Expression

RNA isoPlus^®^ Reagent (Takara, Shiga, Japan) was used to extract total RNA from the liver of adenovirus-administered mice, according to the manufacturer’s protocol. Preparation of first-strand cDNA and quantification of mRNA levels were performed as described previously [11]. The expression levels of ABCC11 were normalized by those of β-actin.

### 4.8. Patients and Sample Collection

This clinical research was conducted according to the Declaration of Helsinki. After obtaining written informed consent, we collected blood samples and surgically removed axillary apocrine glands from axillary osmidrosis patients who visited the plastic surgery Gomi Clinic (Tokyo, Japan) with complaints of axillary odor and underwent the surgical procedure there. Axillary odor levels were scored by sniffing carried out by an experienced medical doctor among the authors, and were classified into six grades (significantly weak, weak, weak-mild, mild, mild-strong, and strong) based on subjective odor ratings. The details of each Japanese patient with AO in this study were as follows: The AO patient carrying *ABCC11* 538GG was a 28-year-old man with “strong” axillary odor; the patient carrying 538GA was a 27-year-old man with “strong” axillary odor; the patient with 538AA was a 23-year-old woman with “significantly weak” axillary odor.

The protocols for sample collection, anonymity, storage, and transportation to RIKEN Yokohama Institute and the University of Tokyo Hospital were approved by the Research Ethics Committee at RIKEN Yokohama Institute (No. H18-8(3)) and the Institutional Review Board of the University of Tokyo (No. 10016). The procedures for *ABCC11* genotyping were approved by the Research Ethics Committee at RIKEN Yokohama Institute. The SmartAmp method-based *ABCC11* genotyping [1,7,8,12] and immunoblotting analysis were carried out at RIKEN Yokohama Institute and the University of Tokyo Hospital, respectively.

### 4.9. Statistical Analysis

All statistical analyses were performed by using EXCEL 2013 (Microsoft Corp., Redmond, WA, USA) with the Statcel3 add-in software (OMS Publishing Inc., Saitama, Japan). Different statistical tests were used for different experiments, as described in the figure legends. The significance of each value was determined when the *p* value was less than 0.05 and 0.01.

## Supplementary Materials

Supplementary materials can be found at www.mdpi.com/1422-0067/18/2/417/s1.

## Abbreviations

ABC	ATP-binding cassette
AO	Axillary osmidrosis
ERAD	Endoplasmic reticulum-associated protein degradation
E_1_S	Estrone sulfate
MRP8	Multidrug resistance-associated protein 8
QPCR	Quantitative polymerase chain reaction
SNP	Single nucleotide polymorphism
WT	Wild-type
