# Supplementary material for: Clinical and Molecular Evidence of ABCC11 Protein Expression in Axillary Apocrine Glands of Patients with Axillary Osmidrosis
Source: Int J Mol Sci. 2017 Feb 15;18(2):417. doi: 10.3390/ijms18020417 (PMC5343951; doi:10.3390/ijms18020417)
Supplement: Supplementary file 1 [file ijms-18-00417-s001.pdf]

Supplementary Materials: Clinical and Molecular Evidence of ABCC11 Protein Expression in Axillary Apocrine Glands of Patients with Axillary Osmidrosis

Yu Toyoda, Tappei Takada, Tsuneaki Gomi, Hiroshi Nakagawa, Toshihisa Ishikawa and Hiroshi Suzuki

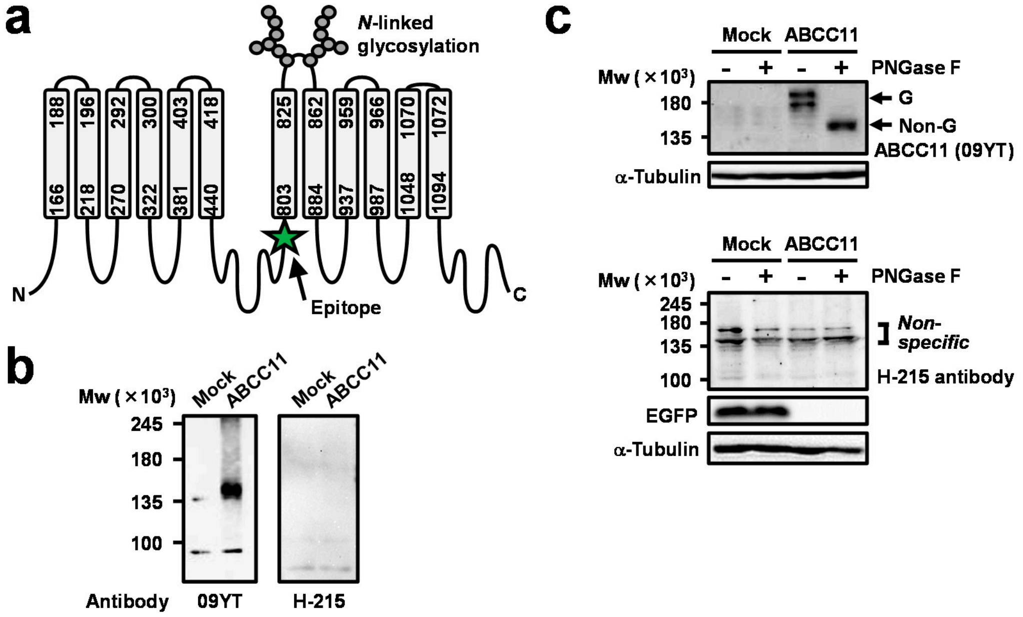

Figure S1. Generation and validation of the anti-ATP-binding cassette C11 (ABCC11) polyclonal antibody. (**a**) Schematic illustration of the ABCC11 protein with the position of the epitope indicated. The putative amino acid sequence in the transmembrane helices was estimated by using the SOSUI program; (**b**,**c**) Validation of the anti-ABCC11 polyclonal antibody (09YT) with immunoblotting using ABCC11-expressing Sf9 cells (**b**) or 293A cells (**c**). In the result from the whole cell lysate of ABCC11-expressing Sf9 insect cells that were derived from our previous study (Toyoda et al., *FASEB J* **2009**, *23*, 2001–2013.), the anti-ABCC11 antibody (09YT) showed a single strong signal with a molecular weight of about 150,000 that corresponds to the non-glycosylated ABCC11 protein. This antibody could recognize both the *N*-glycosylated and non-glycosylated ABCC11 protein exogenously expressed in mammalian 293A cells. The *N*-linked glycosylated forms of ABCC11 (G) were shortened to the non-glycosylated form (Non-G) with peptide *N*-glycosidase F (PNGase F) treatment. On the other hand, H-215—a commercially available antibody—did not detect the ABCC11 protein in our experiments. The enhanced green fluorescent protein (EGFP)-expressing adenovirus was used as a mock virus in (**c**), and adenovirus-mediated protein expression (17 MOI) was confirmed with immunoblotting using an anti-EGFP antibody. α-Tubulin: a loading control.

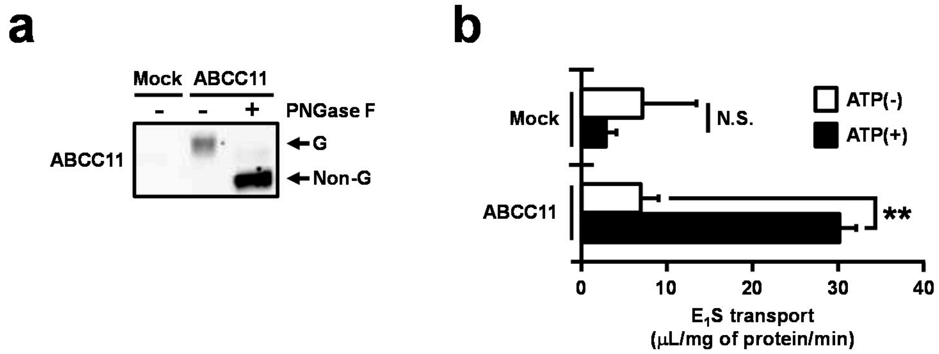

Figure S2. Validation of the ABCC11-expressing adenoviruses. (**a**) Expression of matured-ABCC11 wild-type (WT) protein on the membrane vesicles. ABCC11 WT-expressing or mock membrane vesicles were subjected to immunoblotting analysis with the anti-ABCC11 antibody (09YT). G: glycosylated, Non-G: non-glycosylated form of the ABCC11 protein; (**b**) Estrone sulfate (E_1S) transport activities of ABCC11 in the presence or absence of ATP. E_1S transport into membrane vesicles was measured for 10 min. ATP-dependent incorporation of E_1S was detected in the ABCC11 WT-expressing vesicles, but not in control vesicles. The values are expressed as the mean ± S.D., $n = 3$. Statistical analyses for significant differences were performed according to Student's *t*-test (**, $p < 0.01$; N.S., not significantly different between groups).

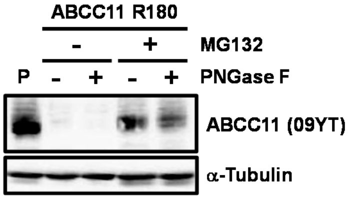

Figure S3. Generation of ABCC11 R180 variant from the adenovirus vector. Thirty-six hours after the infection, ABCC11 R180 variant-expressing Hepa1.6 cells were treated with or without 2 μM MG132 for further 12 hours, then subjected to immunoblotting analysis with the anti-ABCC11 antibody (09YT). P: whole cell lysate of ABCC11 WT-expressing Hepa1.6 cells with both MG132 and PNGase F treatment as a positive control for band position of non-glycosylated ABCC11. α-Tubulin: a loading control. The result showed that ABCC11 R180 protein was not matured as glycoprotein but degraded by MG132-sensitive pathway, which is consistent with the previous report showing that ABCC11 R180 variant is recognized as endoplasmic reticulum-associated protein degradation (ERAD) substrate (Toyoda et al., *FASEB J.* **2009**, *23*, 2001–2013.).
